# Correlation study of polysomnographic parameters, serological markers, and gut microbiota in patients with short-sleep insomnia: a real-world study

**DOI:** 10.3389/fmicb.2026.1850738

**Published:** 2026-08-13

**Authors:** Lu Liu, Hui-Jie Zhou, Hao-Chen Li, Ming-Zhen Li, Yun-Liang Sun, Ji-Wei Zhu, Jing-Lin Wu, Man-Lu Lu, Yan Yu, Fang Han, Lei Pan

**Affiliations:** 1 Department of Respiratory and Critical Care Medicine, Binzhou Medical University, Hospital, Binzhou Medical University, Binzhou, Shandong, China; 2 Department of Respiratory and Critical Care Medicine, The People’s Hospital of Rugao, Nantong, Jiangsu, China

**Keywords:** electroencephalogram, gut microbiota, insomnia, neurotransmitters, polysomnography

## Abstract

**Objective:**

Emerging evidence suggests that the gut microbiota may play a role in sleep regulation via the microbiota–gut–brain axis; however, its relationship with objective sleep parameters and serological markers remains unclear. This study aimed to elucidate the relationship between polysomnographic, serological, and gut microbiota changes in patients with short sleep insomnia.

**Methods:**

Patients with short-sleep insomnia and controls underwent polysomnography. Serum and faecal samples were collected. Serum 5-hydroxytryptamine (5-HT), brain-derived neurotrophic factor (BDNF), melatonin (MT), and orexin A (OXA) were measured by ELISA. Gut microbiota composition was analysed using 16S rRNA sequencing. Spearman’s partial correlation and redundancy analysis (RDA) were performed in the insomnia group with adjustment for relevant confounders.

**Results:**

Compared with controls, insomnia patients showed significantly reduced sleep efficiency (SE), total sleep time (TST), non-rapid eye movement (NREM) and REM sleep duration, and N2 and N3 sleep, accompanied by prolonged sleep latency (SL), REM latency, and wake after sleep onset (WASO) duration and its proportion, as well as increased spontaneous microarousals during total and NREM sleep, and the frequency of sleep stage transitions and awakenings, and the relative power of theta, alpha, and beta waves during NREM sleep and delta, theta, and alpha waves during REM sleep (*p* — 0.05). Serum 5-HT, BDNF, and OXA levels were elevated (*p* — 0.05). While *α*-diversity did not differ, *β*-diversity differed significantly, with increased abundance of *Evtepia*, *Anaerostipes*, and *Blautia_A*, and reduced L-methionine salvage cycle III. Correlation analyses revealed significant associations among gut microbiota, sleep parameters, and serum markers, particularly involving N3 sleep and SL.

**Conclusion:**

Short-sleep insomnia is associated with alterations in sleep parameters, serological indicators, and gut microbiota, suggesting that gut microbiota modulation may represent a potential therapeutic target.

## 1 Introduction

According to the diagnostic criteria of the International Classification of Sleep Disorders, Third Edition (ICSD-3), chronic insomnia disorder is characterised by persistent difficulty initiating sleep, maintaining sleep, or early morning awakening, occurring at least three times per week and lasting for at least three months, despite adequate sleep opportunity, and accompanied by daytime impairments such as fatigue, reduced concentration, or mood disturbance. Chronic insomnia exhibits a high prevalence, seriously affecting people’s quality of life and work. Epidemiological studies have reported a prevalence rate as high as 36.7%, with an increasing trend and a tendency to affect younger age groups (Hou et al., 2023; Wang et al., 2023). Insomnia contributes to heightened societal burdens and significantly impacts individual physical and mental health, with people living with chronic insomnia exhibiting higher risks of hypertension, cardiovascular disease, metabolic syndrome, and depression (Che et al., 2021; Dai et al., 2023; Haghayegh et al., 2023; Pejovic et al., 2024). Therefore, there is an urgent need to establish a multidisciplinary intervention system to address this health challenge.

Short-sleep insomnia is a common subtype of chronic insomnia, characterised primarily by objectively reduced sleep duration (TST — 6 h or SE — 85%, hereafter referred to as short-sleep insomnia) (Johnson et al., 2021). Short-sleep insomnia signifies elevated morbidity and mortality rates. Compared with insomnia with normal objective sleep duration, short-sleep insomnia exhibits stronger associations with stress-activated system (including the hypothalamic–pituitary–adrenal axis (HPA axis), and sympathetic nervous system), and more pronounced tendencies in both genetic predisposition and disease severity (Yang d. F. et al., 2023).

Insomnia can induce low-grade systemic inflammatory response, activate the HPA axis, vagal nerve pathway, immune system, and neurotransmitter release, thereby leading to intestinal dysbiosis, intestinal barrier destruction and altered intestinal permeability, and ultimately affect the composition of intestinal microbiota (Dicks, 2022). Research indicates that the composition of gut microbiota in insomnia patients differs significantly from that in healthy individuals, with a greater abundance of pathogenic bacteria *Streptococcus* in the intestines of insomnia sufferers (Wang Q. et al., 2022). Modulating gut microbiota can improve sleep disorders in patients. Oral administration of *Lactobacillus plantarum PS128* significantly reduced Pittsburgh Sleep Quality Index (PSQI) and Insomnia Severity Index (ISI) scores in insomnia patients (*p* — 0.05), while simultaneously elevating 5-HT levels. It also increased the proportion of delta waves during REM sleep and reduced high-frequency brainwave activity (Ho et al., 2021). A limitation of existing research lies in the fact that the insomnia patients included are mostly screened via scales, without subdividing various subtypes of insomnia. The relationship between short-sleep insomnia and gut microbiota changes remains unclear.

**Figure 1:**
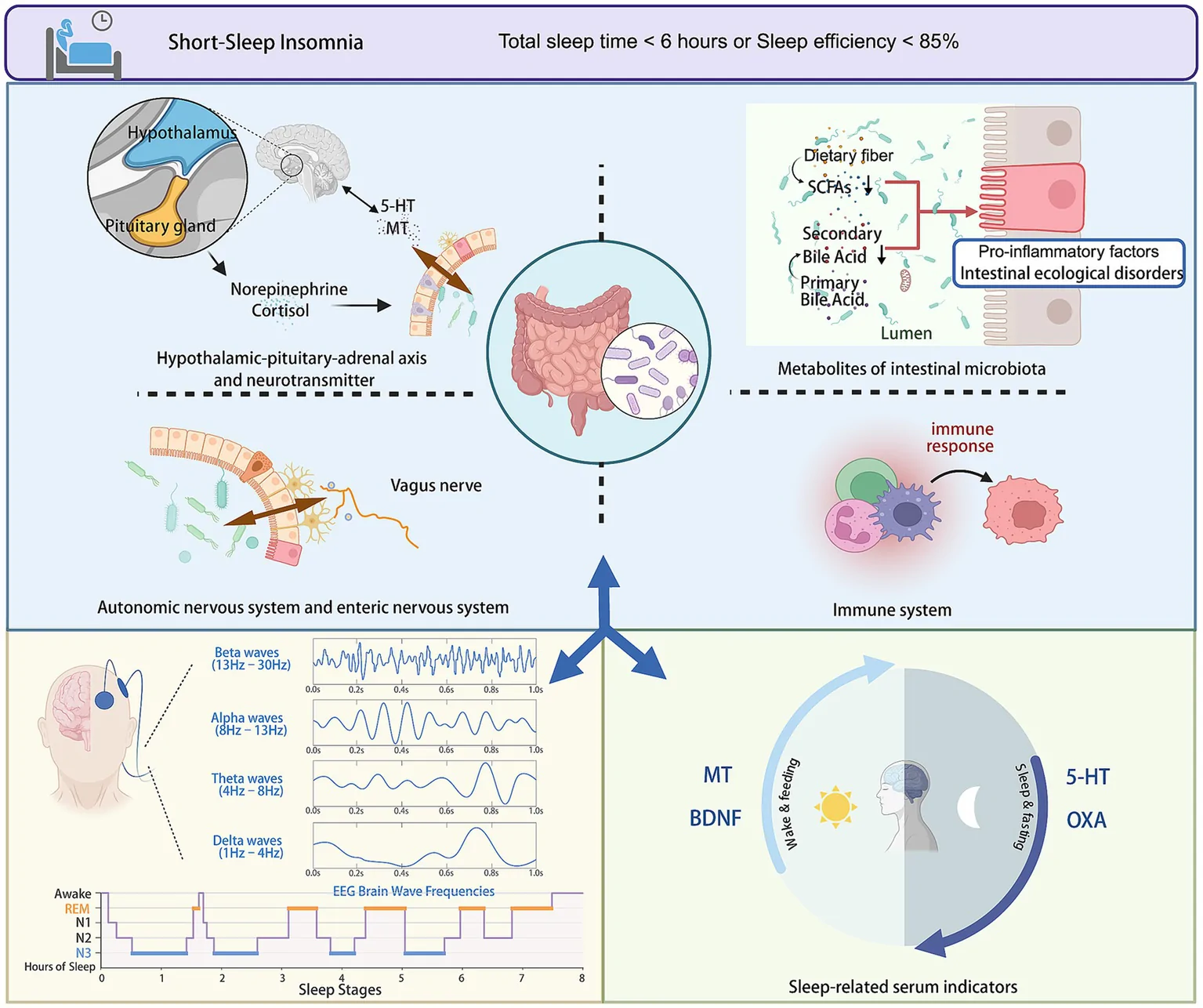
Multi-omics integration analysis reveals a complex network of interactions between gut microbiota, serum factors and sleep architecture in short-sleep insomnia. The study was conducted in patients with short-sleep insomnia. The gut microbiota interacts with insomnia through multiple mechanisms, including the HPA axis, neuroendocrine system, immune system, and microecological imbalance mediated by intestinal metabolites, which can be reflected in the changes of EEG, sleep architecture, and sleep-related serum factors of insomnia patients. 5-HT and OXA belong to the neurotransmitters that promote arousal, while BDNF and MT are neurotransmitters that promote sleep. The release of serum factors was also significantly correlated with sleep rhythm and EEG. Created with BioRender.com.

This study focuses on polysomnographic metrics, characteristic electroencephalogram (EEG) alterations, gut microbiota composition, and sleep-related serum marker levels in short-sleep insomnia patients. It explores the association between these indicators and sleep quality, further elucidating potential mechanisms underlying neurotransmitter-microbiota-sleep interactions to propose novel therapeutic strategies for short-sleep insomnia (Figure 1). Furthermore, this study employs sleep parameters and serological markers as environmental factors, with gut microbiota biomarkers as response variables, conducting RDA. This method assesses the relative importance of ecological factors in shaping differential microbial distributions between insomnia and healthy control groups, thereby providing evidence for targeting gut microbiota biomarker abundance to improve sleep.

## 2 Method

### 2.1 Study population

Fifty-two individuals with insomnia who underwent PSG at the Sleep Disorders Clinic of Binzhou Medical University Affiliated Hospital between November 2023 and December 2024 were selected as the experimental group (Insomnia group). Fifty-nine healthy volunteers without sleep disorders were the control group (Control group). Serum samples were collected from 30 insomnia patients and 19 controls, while faecal samples were obtained from 40 insomnia patients and 33 control subjects. This study was reviewed and approved by the Medical Ethics Committee of Binzhou Medical University Hospital (Ethics Approval No: KT-190), with informed consent obtained from all participants.

Inclusion criteria: ① Insomnia group: Diagnosis of short-sleep insomnia (meeting ICSD-3 criteria); ISI score > 7; PSG demonstrating SE — 85% or TST — 6 h. Control group: ISI score ≤ 7. ② Participants were required to have Hamilton Depression Scale (HAMD) scores ≤ 17 and Hamilton Anxiety Scale (HAMA) scores ≤ 13. ③ Participants were aged ≥ 18 years.

Exclusion criteria: ① Moderate-to-severe obstructive sleep apnea syndrome (OSAS) (AHI ≥ 15 events/h), restless legs syndrome, rapid eye movement sleep behaviour disorder (RBD), sleep groaning, circadian rhythm disorders, or other conditions severely impairing sleep; ② Organic diseases such as diabetes mellitus, coronary heart disease, or hyperthyroidism; ③ Gastrointestinal disorders including irritable bowel syndrome, chronic gastric ulcer, chronic gastritis, or history of *Helicobacter pylori* infection; ④ History of chronic tea or coffee consumption (≥20 g/day), or alcohol intake (>12 g/day) (Koponen et al., 2025); ⑤ Use of oral antibiotic or traditional Chinese medicine, or gastrointestinal endoscopy within the preceding month; ⑥ Pregnancy.

### 2.2 PSG and characteristic EEG analysis methods

A safe and quiet sleep environment was ensured. On the monitoring day, participants were instructed to refrain from napping, taking sleeping pills, or consuming alcohol. Monitoring commenced between 22:00 and 23:00 with lights off, concluding between 06:00 and 07:00 with lights on, ensuring a total monitoring duration of approximately 8 h. PSG must align with the subject’s habitual daily routine and sleep patterns. Monitoring procedure: In accordance with the American Academy of Sleep Medicine (AASM) scoring manual, a ground electrode was placed at the midline of the scalp (Cz). The low frequency filter (LFF) of EEG and electrooculogram (EOG) was set to 0.3 Hz, and the high frequency filter (HFF) was set to 35 Hz. The LFF of electromyogram (EMG) was set to 5 Hz, and the HFF was set to 90 Hz. The LFF of ECG was set to 1 Hz, and the HFF was set to 15 Hz. Leads F4/M1, F3/M2, C3/M2, C4/M1, O1/M2, O2/M1, E1/M2, E2/M1, chin-EMG, and L-EMG were connected. Electrode sites were cleaned, and all impedances were maintained at — 5kΩ before monitoring. Sampling rate was set to 256 Hz. Body position was monitored via video. Subjects underwent consecutive two-night polysomnographic monitoring, with final results derived from the second night. PSG findings were interpreted according to AASM criteria by two experienced sleep technicians. Characteristic EEG components include delta (1–4 Hz), theta (4–8 Hz), alpha (8–13 Hz), and beta (13–30 Hz) waves.

Matlab (MathWorks, Natick, MA, USA) software was employed for characteristic EEG analysis. Patient monitoring results and staging data were imported into Matlab’s EEGLab add-on package. Preprocessing steps included: locating electrode channels, removing redundant electrodes, re-referencing, applying bandpass filtering (0.3–30 Hz), eliminating bad channels and interpolating data, correcting baseline, applying independent component analysis (ICA) to remove ocular, muscular, and cardiac artefacts, and finally segmenting the data. Power spectral density (PSD) of EEG signals was calculated using the Welch method. Relative power in frequency bands during NREM and REM stages was then computed based on the PSD.

### 2.3 Serological indicators

Three millilitres of venous blood were collected from each subject into red-cap tubes (without anticoagulant) between 8:00 and 10:00 a.m. after the second PSG night. The tubes were allowed to stand at room temperature for 1 h for coagulation and separation. The samples were centrifuged at 1000 × *g* for 20 min at 4 °C. The supernatant was transferred into 1.5 mL EP tubes (300 μL per tube) and immediately stored at −80 °C for subsequent analysis. Serum levels of 5-HT, BDNF, MT, and OXA in participants’ samples were measured using ELISA kits (Elabscience, E-EL-0033, E-EL-H0010, E-EL-H2016, and E-EL-H1015).

### 2.4 Gut microbiota sampling and 16S rRNA sequencing

Fresh faecal samples (0.5–1.0 g) were collected from subjects between 7:00 and 8:00 a.m. on the morning following the second night of polysomnographic monitoring. Contamination with urine, blood, or dust was avoided. Using a sterile faecal sampler, the mid-portion of each sample was collected and placed into the sampler. The samples were immediately stored at −80 °C for subsequent analysis. Prior to DNA extraction, uncontaminated faecal samples were thawed on ice. Total microbial DNA was extracted from faecal samples using the Mag-Bind Soil DNA Kit (200) (Omega Bio-Tek, USA). Approximately 500 mg of faecal sample was mixed with glass beads and lysis buffer, and bacterial cells were mechanically disrupted by vortexing. Buffer solution and inhibitor removal reagents were added to remove proteins, polysaccharides, humic-like substances, and insoluble particles. Binding conditions were adjusted to allow DNA to bind to magnetic beads. The magnetic beads were captured using a magnetic rack, the supernatant was discarded, and residual contaminants including proteins and salts were removed by washing. After two rapid washing steps to eliminate trace contaminants, DNA was eluted using Elution Buffer and stored at −20 °C for further use. The extracted DNA’s relative molecular weight and purity were analysed using 0.8% agarose gel electrophoresis. DNA quantification was performed using the NanoDrop NC2000 spectrophotometer (Thermo Fisher Scientific, USA). Equal amounts of DNA from each sample were used for subsequent microbiota analysis. Sequences indicative of microbial community composition and diversity were targeted, such as microbial ribosomal RNA, polymerase chain reaction (PCR) amplification was performed using 16S rRNA V3-V4 region-specific primers. PCR amplification employed specific primers for the bacterial 16S rDNA V3-V4 region: upstream primer 338F (5′-barcode + ACTCCTACGGGAGGCAGCA-3′) and downstream primer 806R (5’-GGACTACHVGGGTWTCTAAT-3′). Amplification products were subjected to 2% agarose gel electrophoresis, followed by recovery of target fragments using a gel extraction kit (AXYGEN, USA). Sequencing libraries were prepared using the TruSeq Nano DNA LT Library Prep Kit (Illumina, USA). Bidirectional sequencing of the 16S rRNA V3-V4 region was performed at 2 × 300 bp read length on the Illumina MiSeq platform using MiSeq Reagent Kit V3 (600 cycles).

After quality control, sequencing data were denoised and chimera sequences were removed using DADA2 to generate an amplicon sequence variant (ASV) feature table. High-quality sequences were clustered into operational taxonomic units (OTUs) at 97% sequence similarity using VSEARCH. To minimise the impact of differences in sequencing depth among samples, the ASV/OTU abundance table was normalised. Prior to *α*- and *β*-diversity analyses, all samples were rarefied to the same sequencing depth. Taxonomic composition was analysed based on relative abundance, and the total bacterial abundance in each sample was classified into different taxonomic levels (phylum, class, order, family, genus, and species). Differential abundance analysis was conducted based on the normalised feature table in combination with appropriate statistical methods. Subsequently, α-diversity indices (Goods_coverage, Shannon, and Simpson indices) were calculated. β-diversity analysis was performed based on the Bray–Curtis distance matrix using Principal Coordinate Analysis (PCoA), and group differences were assessed using the Adonis test. LEfSe analysis was performed based on relative abundance at different taxonomic levels, differential features were identified using the Kruskal–Wallis rank-sum test, and their discriminative effect sizes were evaluated by Linear Discriminant Analysis (LDA). Prior to Orthogonal Partial Least Squares Discriminant Analysis (OPLS-DA), species abundance data were normalised to relative abundance and subjected to appropriate data transformation and scaling to reduce the effects of sequencing depth and highly abundant taxa on the model. Based on the abundance of marker gene sequences in each sample, PICRUSt2 was used to predict the functional composition of microbial communities. Prior to analysis, the ASV abundance table was normalised to relative abundance, and the predicted functional pathways were normalised and analysed based on the MetaCyc database.

### 2.5 Statistical methods

Statistical analyses were performed using R software (version 4.4.2). For continuous variables in independent samples, paired *t*-tests were applied when data met normality assumptions, with results expressed as mean ± standard deviation (SD). When normality was not satisfied, Mann–Whitney *U* tests were used, presenting results as median (Q1, Q3) (to two decimal places). Categorical variables were analysed using chi-square (χ^2^) tests. Standardisation of data employed the *Z*-score/Min-Max method. *p*-values for characteristic EEG features underwent Bonferroni correction. Correlation analysis was performed using Spearman’s partial correlation in R software. Clustering and interactive redundancy analysis (RDA) were performed using R software, with graphical representations generated through GraphPad Prism 9.5, Origin 2024, the OECLOUD platform^1^, and R.

This study employs a between-subject design, Cohen’s d effect size is used to process the effect size (Fabiano et al., 2009). Effect sizes were calculated by subtracting the mean of the control group from the mean of the insomnia group and dividing the result by the pooled standard deviation of the two groups. The 95% confidence intervals (CI) were estimated based on standard error of d. *Post hoc* statistical power was calculated using PASS 15.0 based on the observed effect size and actual sample size (Supplementary Table S1).

Sample size calculations were performed separately for PSG outcomes, serum biomarkers, and microbiota analysis, using PASS 15.0 software. Based on a pilot study, the required sample size for subjects undergoing polysomnographic monitoring was determined. The sample size for the pilot study was established according to Hertzog’s study (Hertzog, 2008), with 20 participants included in both the insomnia group and the control group. No significant differences in age, sex, or BMI were observed between the two groups (*p* ≥ 0.05). The mean ± standard deviation of sleep efficiency in the insomnia group and control group were 76.91 ± 7.22 and 86.19 ± 8.71, respectively. The significance level was set at *α* = 0.05 and the statistical power at 1 − *β* = 0.90. As sleep efficiency in the insomnia group did not follow a normal distribution, the Wilcoxon rank-sum test was applied. Compared with the t-test, the efficiency of the Wilcoxon rank-sum test was estimated to be approximately 95%; therefore, the required sample size for the Wilcoxon test was 1.053 times that required for the t-test (Page et al., 2013). After inflating the sample size by a factor of 1.053 and allowing for a 10% loss to follow-up rate, the minimum required sample size was calculated to be 20 participants in each group. A total of 52 patients with insomnia and 59 control participants were included, which satisfied the sample size requirements.

Based on previous studies, the mean differences in serum 5-HT, MT, BDNF, and OXA between the insomnia group and control group were 37.39 ng/mL, −9.3512 pg/mL, −7.01 pg/mL, and 313.21 pg/mL, respectively. The standard deviations of serum 5-HT, MT, BDNF, and OXA in the insomnia group were 13.28 ng/mL, 8.51 pg/mL, 3.35 pg/mL, and 97.39 pg/mL, respectively, while those in the control group were 13.15 ng/mL, 9.38 pg/mL, 4.22 pg/mL, and 60.18 pg/mL, respectively (Can and Tuzer, 2023; Gong et al., 2023; Gultuna et al., 2024). The significance level was set at *α* = 0.05 and the statistical power at 1 − *β* = 0.80. After allowing for a 10% loss to follow-up rate, the minimum required sample size was calculated to be 18 participants in each group. Serum samples were collected from subjects who had completed PSG, and a total of 30 patients with insomnia and 19 control participants were included, which was sufficient for statistical analysis.

Based on the pilot study, 20 participants who had completed PSG were included in both the insomnia group and the control group. The α-diversity index Faith’s PD in the insomnia group and control group were 76.01 ± 27.78 and 48.38 ± 18.38, respectively. The significance level was set at *α* = 0.05 and the statistical power at 1 − *β* = 0.90. As Faith’s PD did not follow a normal distribution, a sample size equivalent to 1.053 times that required for the t-test was used. After inflating the sample size by a factor of 1.053 and allowing for a 10% loss to follow-up rate, the minimum required sample size was calculated to be 20 participants in each group. Faecal samples were collected from subjects who had completed PSG, and a total of 40 patients with insomnia and 33 control participants were included (two control samples were excluded due to contamination), meeting the calculated sample size requirement.

## 3 Results

### 3.1 Basic information and clinical characteristics of short-sleep insomnia patients and the control group included in the study

No significant differences were observed between the insomnia and control groups in terms of age, gender, BMI, and AHI (*p*>0.05), as shown in Table 1.

**Table 1:** Basic information and clinical characteristics of the insomnia and control groups.

Classification		Gender (woman)	Age, year	BMI, kg/m^2^	AHI, events/h	Use of medication (yes)	Duration of insomnia ≤ 3 months (yes)
Polysomnographic parameters	Insomnia Group	52(31)	50.50 (37.25, 50.90) ^#^	23.60(22.12, 26.01)	4.75(1.43, 10.60)	52(21)	52(18)
	Control Group	59(31)	46.00 (39.00, 59.00) ^#^	25.26(22.82, 27.20)	6.10 (2.50, 10.90)		
	*χ^2^*/*t*/*Z*	0.560	1.550	−1.940	−1.232		
	*p*	0.454	0.124	0.053	0.218		
Serological indicators	Insomnia Group	30(17)	53.50(36.75, 59.25)	23.60(22.54, 25.17)	4.55(1.65, 10.93)	30(14)	30(7)
	Control Group	19(7)	42.00(37.00, 50.00)	25.86(23.42, 27.40)	6.20 (0.90, 12.60)		
	*χ^2^*/*t*/*Z*	1.829	−1.848	−1.950	−0.821		
	*p*	0.176	0.065	0.051	0.412		
Gut microbiota	Insomnia Group	40(25)	52.50 (38.50, 59.00) ^#^	23.65(22.60, 26.33)	4.85(1.23, 10.88)	40(18)	40(12)
	Control Group	33(17)	47.00 (39.50, 54.00) ^#^	25.26(22.96, 27.56)	5.90 (2.75, 12.65)		
	*χ^2^*/*t*/*Z*	0.890	1.550	−1.680	−1.114		
	*p*	0.345	0.126	0.093	0.265		

BMI, body mass index; AHI, Apnoea-Hypopnoea Index; ^#^: The data are normally distributed.

### 3.2 Analysis of polysomnographic parameters in short-sleep insomnia patients and the control group

Analysis of polysomnographic parameters for both groups (Table 2) showed no significant difference in total recording time between the insomnia and control groups (*p* = 0.371). The insomnia group exhibited significantly reduced SE, TST, and NREM sleep stages compared to the control group (*p*—0.001). WASO and its proportion were significantly increased (*p*—0.001), while SL was markedly prolonged (*p*—0.001). The insomnia group showed significantly shorter stages of REM, N2, and N3 compared with the control group (*p*—0.01). However, the proportions of REM, N2, and N3 sleep stages in the insomnia group did not differ significantly from those in the control group (*p*>0.05). The frequency of spontaneous micro-awakenings during total sleep and NREM sleep, and the frequency of sleep stage transitions were significantly higher in the insomnia group than in the control group (*p*—0.01). Compared with the control group, the insomnia group exhibited significantly increased REM latency and the frequency of awakening from sleep (*p*—0.05), with a notable trend towards increased REM latency proportion (*p* = 0.062).

**Table 2:** Polysomnographic data for the insomnia and control groups.

Polysomnographic parameters	Overall (*N* = 111)	Insomnia Group (*N* = 52)	Control Group (*N* = 59)	*t*/*Z*	*p*
SE, %	79.70 (69.10, 87.90)	75.00 (61.93, 80.35)	86.70 (78.60, 92.50)	−5.393	—0.001***
TST, min	379.00 (316.00, 416.00)	347.00 (304.75, 383.75)	404.00 (374.00, 427.00)	−4.651	—0.001***
TRT^#^, min	477.00 (452.50, 495.50)	480.00 (458.00, 495.88)	470.00 (446.00, 493.00)	0.898	0.371
REM sleep^#^, min	71.50 (54.53, 91.00)	66.76 (47.75, 83.38)	77.50 (58.00, 97.00)	−2.838	0.005**
Proportion of REM sleep, %	19.50 (16.20, 23.40)	19.40 (15.60, 23.15)	19.50 (16.20, 24.10)	−0.473	0.636
NREM sleep, min	300.50 (258.00, 336.00)	277.75 (242.88, 307.38)	326.50 (280.50, 344.50)	−4.293	—0.001***
Proportion of NREM sleep, %	80.50 (76.60, 83.80)	80.60 (76.85, 84.40)	80.50 (75.90, 83.80)	−0.452	0.651
N1 sleep^#^, min	46.50 (29.00, 66.50)	48.75 (29.00, 68.38)	46.00 (29.00, 65.50)	0.400	0.690
Proportion of N1 sleep, %	13.50 (8.10, 19.70)	14.05 (9.35, 21.10)	12.60 (7.40, 19.70)	−1.569	0.117
N2 sleep^#^, min	164.50 (130.00, 198.00)	150.25 (115.13, 176.00)	179.00 (145.00, 211.00)	−3.300	0.001**
Proportion of N2 sleep^#^, %	45.00 (38.30, 51.70)	44.40 (38.38, 51.50)	45.70 (38.30, 52.20)	0.604	0.547
N3 sleep^#^, min	73.50 (48.50, 99.00)	69.00 (33.50, 92.50)	82.50 (54.00, 105.00)	−2.900	0.005**
Proportion of N3 sleep, %	20.20 (13.00, 27.20)	19.45 (11.73, 27.65)	22.00 (14.30, 25.90)	−1.433	0.152
WASO, min	54.50 (23.50, 94.50)	78.75 (53.38, 131.13)	29.00 (16.00, 63.00)	−4.743	—0.001***
Proportion of WASO, %	14.41 (6.18, 27.81)	20.06 (14.52, 44.81)	7.41 (3.98, 15.76)	−5.070	—0.001***
SL, min	24.50 (11.00, 40.00)	31.75 (14.13, 50.38)	18.00 (7.50, 31.00)	−3.082	0.002**
REM latency, min	93.25 (67.38, 136.25)	107.00 (74.50, 157.50)	84.00 (64.00, 115.00)	−2.284	0.022*
Proportion of REM latency, %	24.40 (18.02, 37.56)	28.97 (20.75, 45.31)	20.05 (16.54, 33.56)	−1.870	0.062
The frequency of spontaneous microarousals during total sleep, events/h	3.00 (1.20, 7.30)	5.20 (1.70, 9.90)	2.30 (1.00, 4.50)	−2.867	0.004**
The frequency of spontaneous microarousals during REM sleep, events/h	2.50 (0.00, 5.28)	3.10 (0.00, 7.60)	2.10 (0.60, 3.70)	−0.795	0.426
The frequency of spontaneous microarousals during NREM sleep, events/h	3.00 (1.00, 7.00)	5.20 (1.60, 10.68)	2.20 (0.90, 4.80)	−2.852	0.004**
Awakening from sleep^#^, events	17.00 (11.00, 23.00)	18.50 (10.25, 23.75)	16.00 (11.00, 23.00)	0.489	0.626
The frequency of awakening from sleep^#^, events/h	2.94 (2.03, 3.83)	3.27 (2.13, 4.19)	2.51 (1.55, 3.52)	2.413	0.017**
Sleep stage transitions^#^, events	103.00 (76.00, 124.00)	107.50 (75.25, 139.00)	102.00 (77.00, 120.00)	−0.089	0.930
The frequency of sleep stage transitions, events/h	16.50 (12.13, 20.74)	19.06 (14.33, 22.61)	15.29 (11.12, 18.54)	−2.624	0.009***

BMI, body mass index, AHI: Apnoea-Hypopnoea Index; ^#^: The data are normally distributed; *: *p* — 0.05, **: *p* — 0.01, ***: *p* — 0.001; Total Sleep Time (TST): Total time from the appearance of the subject’s first frame of sleep-related staging to the end of the last frame of sleep-related staging minus time awake; Total Recording Time (TRT): Total time from light off recording frame to light on recording frame; Sleep Efficiency (SE): Percentage of TST and TRT; Rapid Eye Movement (REM): The proportion of REM sleep refers to the percentage of the REM sleep and TST; Non-Rapid Eye Movement (NREM): NREM sleep, which includes N1, N2, and N3 stages. The proportion of NREM sleep and each stage refers to the percentage of the duration of NREM sleep and each stage and TST; Wake After Sleep Onset (WASO): The time awake from sleep until the end of recording. The proportion of WASO refers to the percentage of WASO and TST; Sleep Latency (SL): From the onset of recording to the time of the first appearance of sleep-related stages; REM latency: The time from falling asleep to the first appearance of REM sleep. The proportion of REM latency refers to the percentage of REM latency and TST; Spontaneous microarousals: Spontaneous microarousals during sleep. The frequency of spontaneous microarousals refers to the number of such events that occur spontaneously within each hour of sleep stage; awakening from sleep (frequency, per hour): The frequency of transitions from REM or NREM sleep to the wakefulness phase during the sleep process. The frequency of awakening from sleep refers to the number of times one awakens per hour during sleep; Sleep stage transitions (frequency, per hour): The number of transitions between sleep stages during the sleep. The frequency of sleep stage transition refers to the number of transitions per hour during sleep.

**Figure 2:**
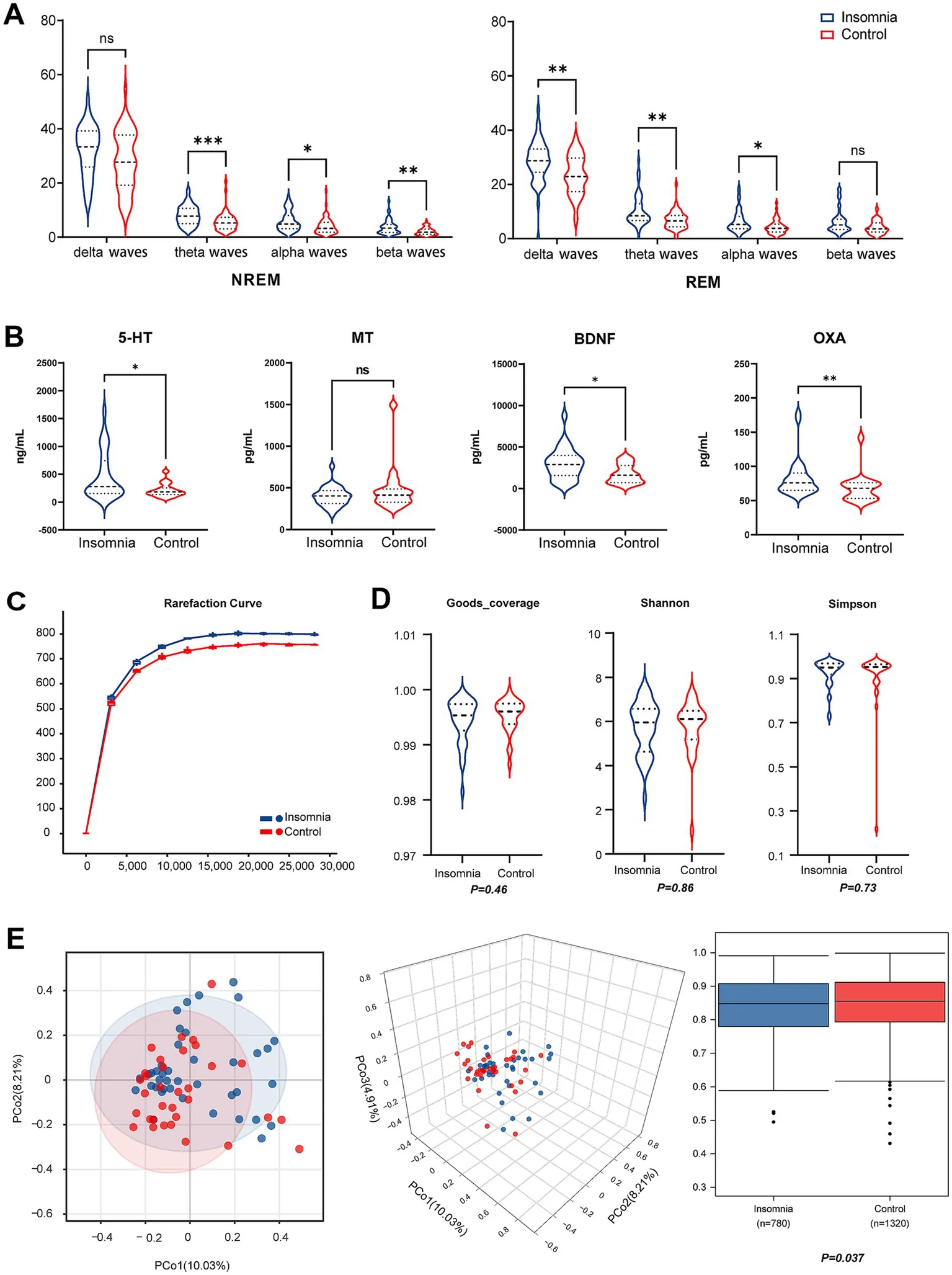
Analysis of the relative power of characteristic EEG, serum biomarkers, and gut microbiota diversity between insomnia patients and controls. **(A)** Analysis of the relative power of characteristic EEG between the insomnia and control groups. **(B)** Analysis of serum biomarkers between the insomnia and control groups. **(C)** The sparse curves for Chao1. **(D,E)** Analysis of *α* and *β*-diversity in gut microbiota. *: *p* — 0.05, **: *p* — 0.01, ***: *p* — 0.001, ns: insignificant.

Analysis of the relative power of characteristic EEG components (Figure 2A) showed that theta, alpha, and beta waves during NREM sleep, as well as delta, theta, and alpha waves during REM sleep, exhibited significantly higher relative power in the insomnia group than in the control group (Bonferroni-corrected, *p*—0.05) (Supplementary Table S2). Characteristic EEG components demonstrated a progressive decline in relative power across frequency bands.

### 3.3 Analysis of serological indicators in short-sleep insomnia patients and controls

Serum levels of 5-HT and BDNF in the insomnia group were significantly higher than those in the control group (*p* — 0.05). Furthermore, OXA levels were also markedly elevated in the insomnia group (*p* — 0.01). Compared with the control group, MT levels in the insomnia group showed a decreasing trend but did not exhibit significant differences (*p* = 0.315) (Figure 2B; Supplementary Table S3).

ROC curves were constructed using 5-HT, BDNF, and OXA to further analyse the diagnostic sensitivity of these markers for insomnia. The areas under the ROC curves for 5-HT, BDNF, and OXA were 0.676 (95%CI, 0.514–0.818), 0.702 (95%CI, 0.544–0.859), and 0.721(95%CI, 0.575–0.867), respectively (*p*—0.05), suggesting good discriminatory power for insomnia (Supplementary Figure S1).

### 3.4 Gut microbiota analysis of short-sleep insomnia patients versus controls

#### 3.4.1 Sequencing quality assessment

The rarefaction curves for Chao1 in this study exhibited a levelling trend, indicating stable sequencing results that adequately reflect the diversity present in the current samples (Figure 2C).

#### 3.4.2 Diversity analysis

*α*-diversity analysis revealed that the Goods_coverage indices for all samples exceed 0.98, indicating sufficient species richness. Analysis using Shannon and Simpson indices showed no significant differences in α-diversity (*p*>0.05) (Figure 2D). As shown in Figure 2E, *β*-diversity within the insomnia group exhibited a more pronounced intragroup variation compared with the control group. Apparent intragroup clustering was observed for both groups, with some overlap between them. However, statistical analysis using Adonis indicates differing degrees of intragroup clustering, revealing significant intergroup differences (*Pr* = 0.037) (Supplementary Table S4).

#### 3.4.3 Compositional analysis and differentiating species

**Figure 3:**
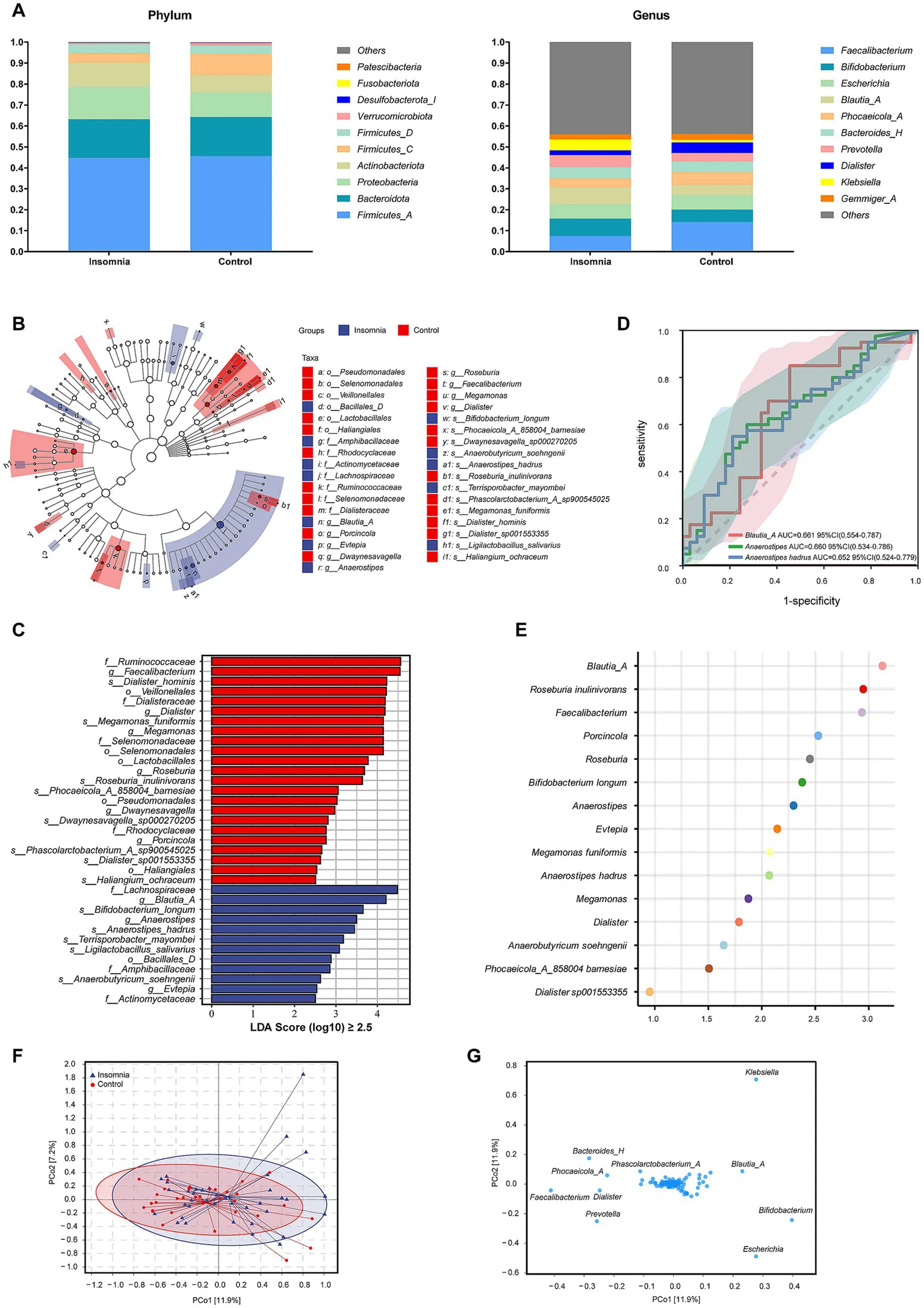
Species composition and differential species analysis of gut microbiota between insomnia patients and controls. **(A)** Species composition analysis of gut microbiota (phylum and genus levels) between the insomnia and control groups. **(B)** LEfSe taxonomic cladogram for insomnia and control groups, illustrating the hierarchical relationship from phylum to genus (from inner to outer circles) within sample communities. Node size corresponds to the average relative abundance of the taxonomic unit; hollow nodes indicate no significant intergroup difference, while coloured nodes denote significant intergroup differences and higher abundance within the colour-coded sample group. **(C)** LDA bar chart, where longer bars indicate greater differential abundance of the taxonomic unit. **(D)** ROC curve for gut microbiota markers, with shaded areas beneath the curve representing the 95% confidence intervals. **(E)** RF analysis of gut microbiota, where higher values on the x-axis denote greater significance of the bacterial genus (species level) for group differentiation. **(F,G)** OPLS-DA plot and loading plot. The selection criterion for principal component analysis at the genus level filters out components unrelated to grouping, displaying only those showing compositional differences between groups. Species similarity is evaluated using the abundance matrix; genera closer to the loading plot’s upper-right corner contributed more significantly to OPLS-DA-detected differences.

As shown in Figure 3A, at the phylum level, *Firmicutes_A*, *Bacteroidota*, and *Proteobacteria* were the most abundant phyla, accounting for 44.75, 18.43, and 15.62% respectively, in the insomnia group, and 45.61, 18.67, and 11.34%, respectively, in the control group. (Species composition at the class, order, family, and species levels is shown in Supplementary Figure S2.)

LEfSe identified gut microbiota with LDA scores ≥ 2.5 when comparing the insomnia group with the control group (Kruskal-Wallis test, *p* — 0.05; *FDR*—0.05), and intergroup differences were further tested using the Wilcoxon test (*p* — 0.05). A total of 35 gut microbial taxa exhibited significant differences, encompassing six orders, seven families, nine genera, and thirteen species. At the order level, one order was elevated in the insomnia group and five in the control group. At the family level, three were elevated in the insomnia group and four in the control group. At the genus level, three showed increased abundance in the insomnia group, namely *Evtepia*, *Anaerostipes*, and *Blautia_A*, while six were significantly elevated in the control group, namely *Faecalibacterium*, *Dialister*, *Megamonas*, *Roseburia*, *Dwaynesavagella*, and *Porcincola*. Figure 3B illustrated the distribution of significantly different gut microbiota and their taxonomic relationships. The most abundant significantly different gut microbiota belong to the *Lachnospiraceae* family, exhibiting the highest relative abundance in the insomnia group. Notably, while the abundance of *Lactobacillales* was considerably higher in the control group than in the insomnia group, the abundance of *Ligilactobacillus_salivarius* within this taxonomic unit was significantly elevated in the insomnia group. Figure 3C displayed the differentially abundant taxa in bar chart format. The most specific taxa in the insomnia group and control group were *Lachnospiraceae* and *Ruminococcaceae*, respectively. Random forest (RF) analysis (genus and species levels) indicated that *Blautia_A*, *Roseburia_inulinivorans*, and *Faecalibacterium* constituted the most significant bacterial groups distinguishing the insomnia group from the control group (Figure 3E).

Receiver Operating Characteristic (ROC) curves were constructed for gut microbiota markers identified by LEfSe analysis (Figure 3D). A total of nine gut microbiota species demonstrated diagnostic value for insomnia (*p*—0.05). Among these, *Blautia_A*, *Anaerostipes*, and *Anaerobutyricum_soehngenii* exhibited an area under the ROC curve (AUC) > 0.65 (*p*—0.05) at both genus and species levels, indicating favourable discriminatory ability for insomnia.

OPLS-DA indicated that genera exhibiting intergroup differences in abundance show more pronounced clustering in the insomnia group compared with the control group, demonstrating significant differences between the two groups. Concurrently, the loading plot revealed the top ten genera by abundance contribution, with *Klebsiella* making the most significant differential contribution to the OPLS-DA analysis across both groups (Figures 3F,G).

#### 3.4.4 Association network analysis

**Figure 4:**
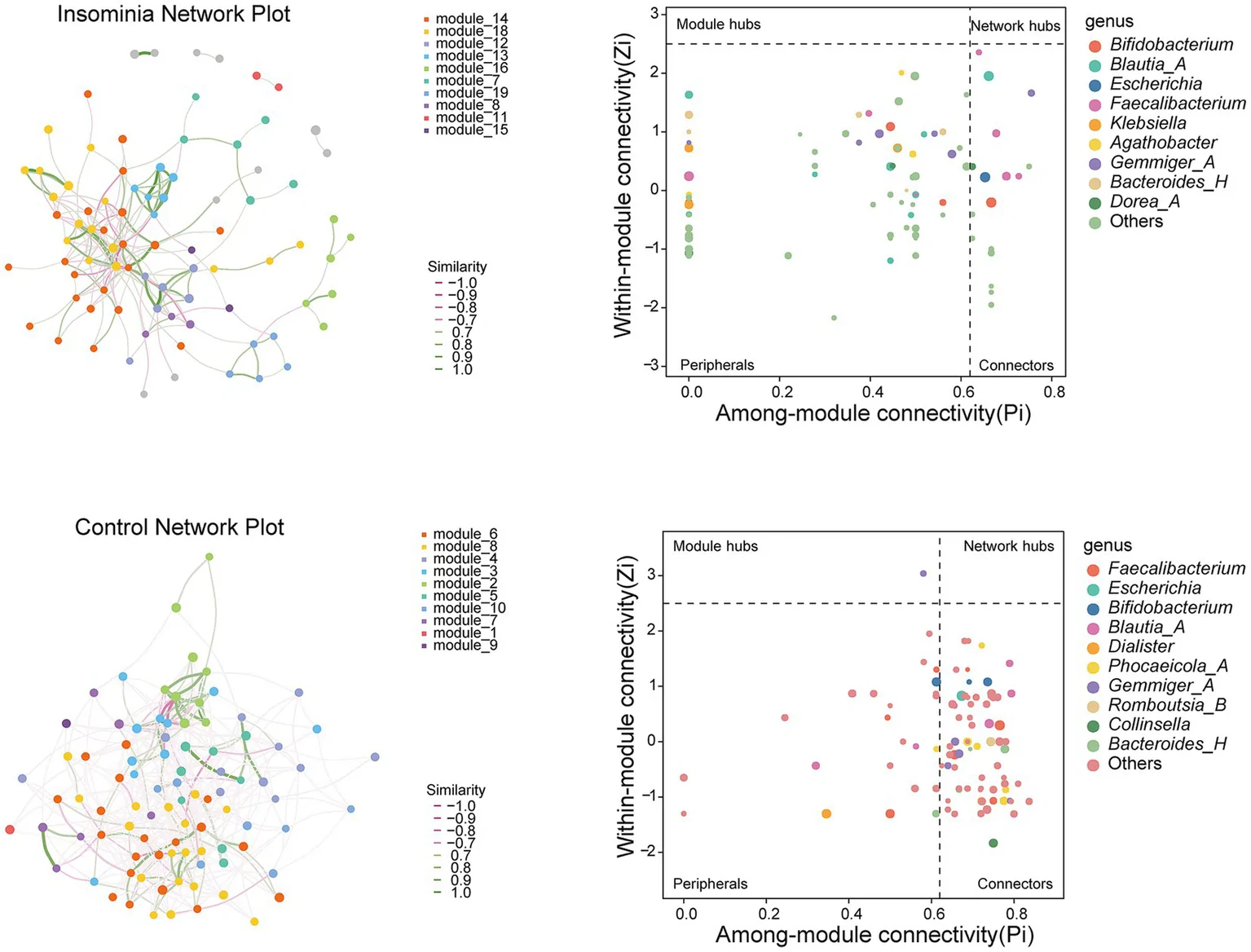
Association networks and ZiPi plots for insomnia and control groups. Nodes represent the sample’s top 90 most abundant Amplicon Sequence Variants (ASVs), with larger nodes indicating higher relative abundance. Modules with the top ten number of nodes are colour-coded. Edges between nodes indicate correlations between the connected nodes, with green denoting positive correlations and red denoting negative correlations; the colour intensity reflects the strength of the correlation. The diagram on the right depicts the Pi and Zi values of nodes in the network along the *x* and *y* axes, respectively. The Zi value indicates a node’s connectivity within its module, measuring its clustering within that module. The Pi value reflects a node’s connectivity with other modules across the entire network, measuring its bridging role between different modules. Microbial communities positioned closer to the upper right corner play a more critical role within their respective samples.

As illustrated in Figure 4, compared with the control group, microbial connections in the insomnia group were more concentrated among a subset of bacterial communities. Conversely, the control group’s gut microbiota featured more node connections and more evenly distributed modules, suggesting stronger overall cooperative relationships within this group. *Gemmiger_A* played a pivotal role in the control group, indicating that alterations in its abundance may induce shifts in the distribution of the control group’s microbiota. No comparable network hubs or nodes with similar influence were identified among the genera in the insomnia group.

#### 3.4.5 Functional predictive analysis

**Figure 5:**
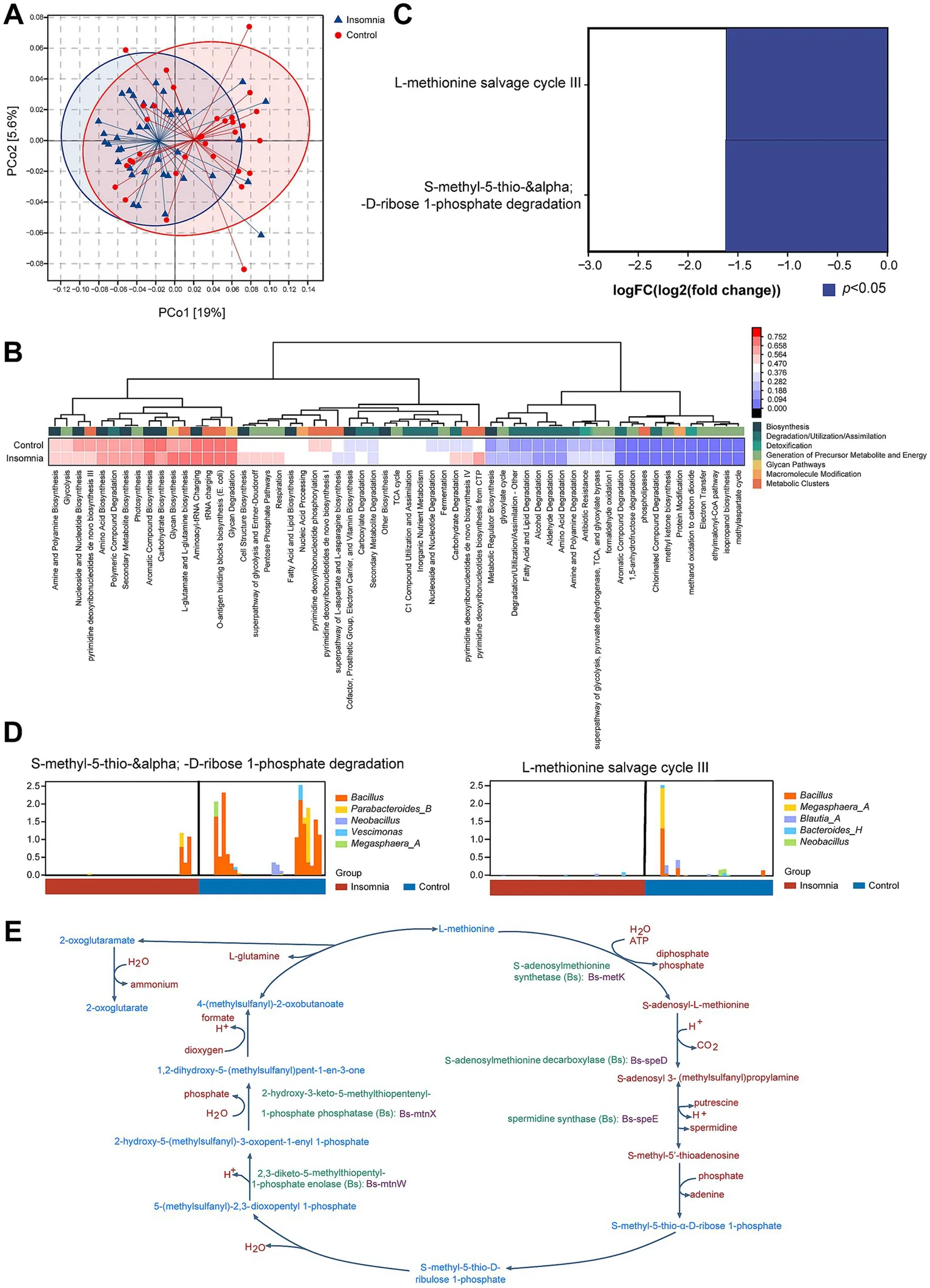
Functional prediction analysis of gut microbiota. **(A)** PCoA plot of predicted functional units in gut microbiota. **(B)** Abundance of MetaCyc second-level functional pathways. **(C)** Analysis of differentially metabolic pathways between the insomnia and control groups; positive values on the logFC (log2(fold change)) axis indicate upregulation in insomnia relative to control, negative values indicate downregulation. **(D)** Major encoding microbiota composition (genus level) for differentially expressed metabolic pathways in gut microbiota. **(E)** Upstream and downstream features of differential metabolic pathways; the figure illustrates the L-methionine salvage cycle III pathway; blue text denotes the S-methyl-5-thio-alpha-D-ribose 1-phosphate degradation pathway.

As demonstrated by the PCoA results for gut microbiota functional prediction units, intragroup variation was markedly greater in the control group than in the insomnia group. Samples from the insomnia and control groups exhibited significantly different projection distances on the coordinate axes (intergroup differences analysed using the Adonis statistical method, *p* = 0.004), indicating substantially distinct functional compositions between the two groups within this dimension (Figure 5A; Supplementary Table S5).

Metabolic pathway prediction analysis, as depicted in Figure 5B, revealed that the insomnia group exhibits compositional differences compared with the control group in L-methionine salvage cycle III and S-methyl-5-thio-alpha-D-ribose 1-phosphate degradation (*FDR* — 0.05, adj*P*-value = 0.013) (Figure 5C). Genes encoding the L-methionine salvage cycle III are predominantly represented at the genus level by *Bacillus* and *Megasphaera_A*. Genes involved in S-methyl-5-thio-alpha-D-ribose 1-phosphate degradation are predominantly encoded by *Bacillus*, *Parabacteroides_B*, and *Neobacillus* at the genus level. These dominant microbial groups account for most functional activity in the differentially regulated metabolic pathways, and are significantly enriched in the control group (Figure 5D). Figure 5E primarily displays genes, enzymes, and metabolites associated with *Bacillus*. Genes linked to *Bacillus* include those such as *speE-G275*, *mteK BSU30550*, and *mteK BSU29010*.

### 3.5 Correlation analysis of polysomnographic parameters, serological markers, and gut microbiota in patients with short-sleep insomnia

#### 3.5.1 Spearman’s partial correlation analysis

**Figure 6:**
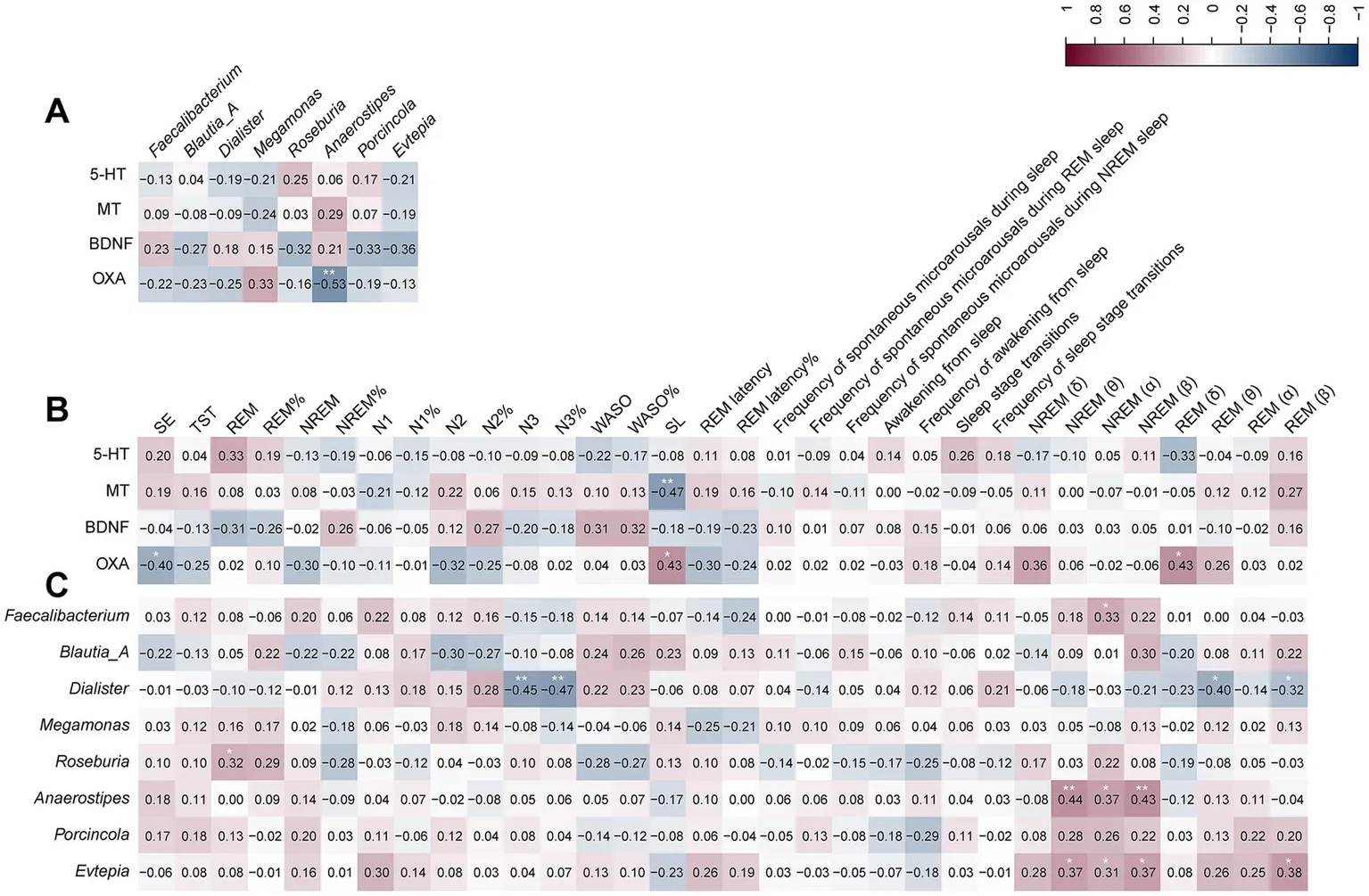
Spearman’s partial correlation analysis of polysomnographic parameters, serological indicators, and gut microbiota in insomnia patients. **(A)** Spearman’s partial correlation analysis of serological indicators and gut microbiota in insomnia patients. **(B)** Spearman’s partial correlation analysis of polysomnographic parameters and serological indicators in insomnia patients. **(C)** Spearman’s partial correlation analysis between polysomnographic parameters and gut microbiota in insomnia patients. *: *p* — 0.05, **: *p* — 0.01, ***: *p* — 0.001; colour intensity and boxed numbers indicate correlation coefficient magnitude.

This study controlled for confounding factors by including age, gender, BMI, AHI, duration of insomnia, and medication use as covariates. Specifically, Spearman’s partial correlation analysis was conducted to examine associations between sleep parameters and serological indicators in insomnia patients who had completed PSG and serum collection. As depicted in Figure 6B, MT exhibits a significant negative correlation with SL (*R* = −0.474, *p* = 0.009). OXA shows a negative correlation with SE (*R* = −0.396, *p* = 0.031), and a significant positive correlation with both SL (*R* = 0.433, *p* = 0.018) and relative power of delta waves during REM sleep (*R* = 0.428, *p* = 0.019). Spearman’s partial correlation analysis for insomnia patients with complete microbiota and serum samples (Figure 6A) indicates a significant negative correlation between OXA and *Anaerostipes* (*R* = −0.528, *p* = 0.009). Spearman’s partial correlation analysis for insomnia patients with complete microbiota and polysomnographic data, as shown in Figure 6C, reveals that *Anaerostipes* positively correlates with the relative power of theta and beta waves during NREM sleep, while *Dialister* shows a significant negative correlation with N3 sleep and its proportion (*p* — 0.01). *Roseburia* positively correlates with REM sleep; *Anaerostipes* positively correlates with relative power of alpha waves during NREM sleep; *Evtepia* positively correlates with relative power of theta, alpha, and beta waves during NREM sleep, as well as relative power of beta waves during REM sleep; *Dialister* shows a negative correlation with relative power of theta and beta waves during REM sleep (*p*—0.05); *Faecalibacterium* exhibits a significant positive correlation with relative power of alpha waves during NREM sleep (*R* = 0.326, *p* = 0.040).

#### 3.5.2 Interactive redundancy analysis (RDA)

**Figure 7:**
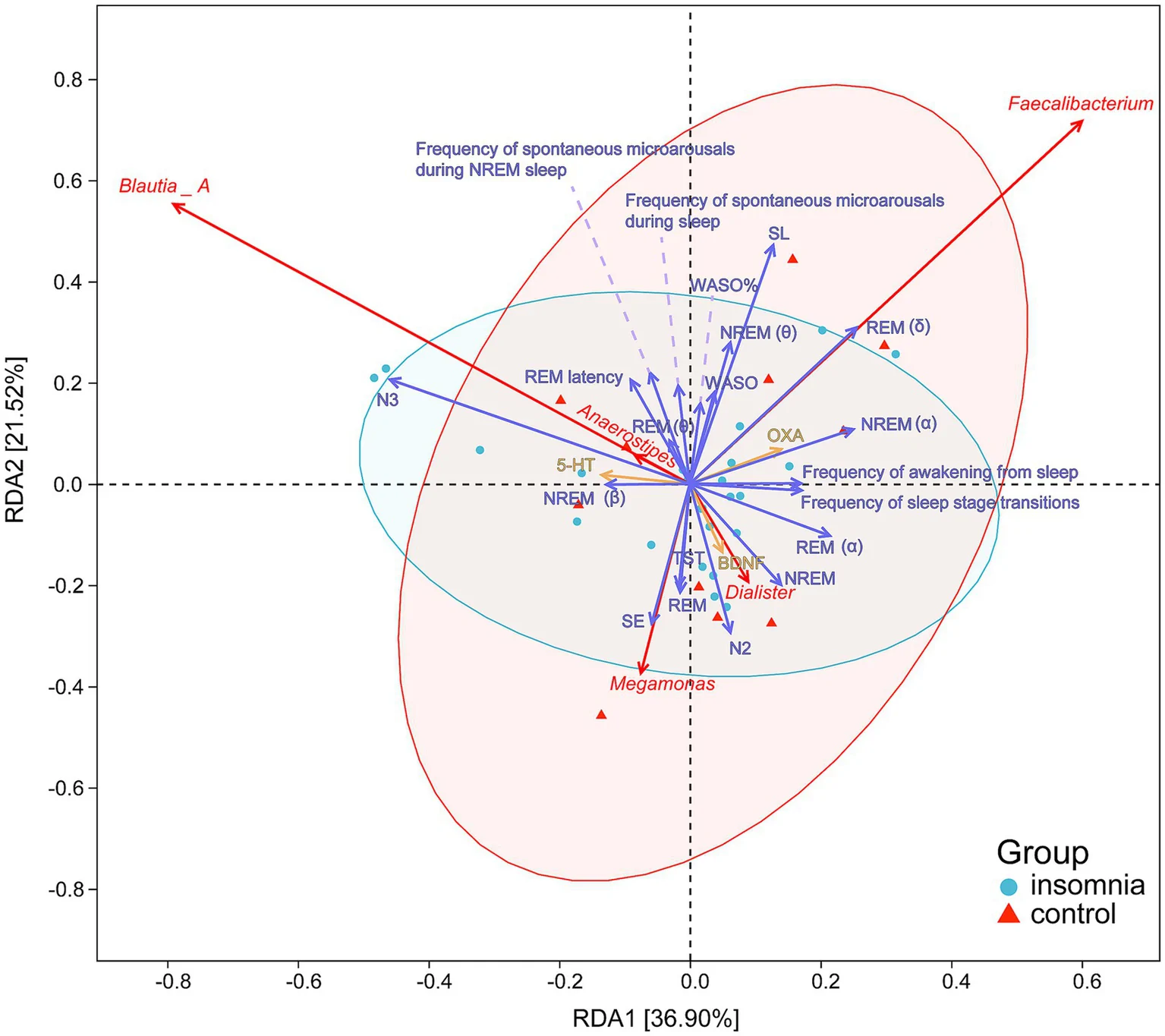
RDA of interaction between insomnia-related sleep parameters, serum indicators and gut microbiota biomarkers. Ellipses denote 95% confidence intervals for sample distributions across groups. Different colours distinguish distinct biomarkers. Dashed lines extend solid lines solely for labelling convenience without additional significance; solid line length indicates correlation strength.

This analysis included subjects from both the insomnia and control groups who concurrently completed PSG, serum, and faecal sampling. Polysomnographic parameters and serum indicators were uniformly standardised. Sleep parameters and serum served as environmental factors, while gut bacterial genus markers acted as response factors in the RDA. As illustrated in Figure 7, the explanatory variables, composed of polysomnographic parameters and serological indicators, collectively explain 58.42% of the sample distribution variance. This indicated a strong correlation between these data and gut microbiota markers, alongside interactions among environmental factors. The figure displayed the top five genera most significant for gut microbiota distribution: *Blautia_A*, *Faecalibacterium*, *Dialister*, *Megamonas*, and *Anaerostipes*. Following 999 Monte Carlo permutations, statistically significant correlations were observed between N3 sleep and SL and gut microbiota markers distribution in both the insomnia and control groups (N3 sleep: *R^2^* = 0.2344, *p* = 0.014; SL: *R^2^* = 0.1857, *p* = 0.044).

## 4 Discussion

### 4.1 Differences in polysomnographic parameters between short-sleep insomnia patients and controls

The results of the PSG in this study were consistent with the sleep characteristics of short-sleep insomnia patients, which may be associated a series of insomnia-related physiological and pathological alterations. Research has indicated that individuals with reduced TST are characterised by disrupted inflammatory homeostasis, heightened sympathetic nervous system activity, and altered gut microbiota composition in short sleepers (Nôga et al., 2024). Conversely, these factors may also influence sleep duration (Besedovsky et al., 2019; Dicks, 2022; Tai et al., 2023). Sleep deprivation may induce low-grade inflammation by impairing clearance of pathogen-associated molecular patterns and damage-associated molecular patterns (Boespflug and Iliff, 2018; Brzecka et al., 2018), whilst pro-inflammatory and anti-inflammatory responses themselves exacerbate sleep disturbances (Besedovsky et al., 2019). Additional research has indicated that, compared with non-insomnia individuals with objectively shortened sleep duration, short-sleep insomnia patients exhibit markedly elevated cortisol levels and sympathetic nervous activity (Vgontzas et al., 2001). This suggests a physiological tendency towards excessive arousal during sleep in short-sleep insomnia patients, a finding correlated with increased microarousals and frequency of sleep stage transitions.

In the present study, the relative power of beta waves during NREM sleep in insomniacs elevated significantly, suggesting increased cortical arousal and consequent impairment of sleep continuity (Riemann et al., 2010). Zhao et al. (2021)’s research demonstrated increased relative power of beta waves during NREM sleep in insomnia patients, consistent with the present findings. Delta waves facilitate deep cerebral rest and recovery (Léger et al., 2018). During REM sleep, insomnia patients exhibited significantly elevated relative power of delta waves, while the relative power of beta waves showed no significant difference. This may relate to compensatory increases in REM sleep induced by chronic sleep deprivation (Schwartz and Mong, 2011). Research has found that following prolonged sleep deprivation, rats exhibit a significant increase in the frequency of REM sleep episodes relative to NREM sleep (Franken, 2002). This may explain the differing patterns of delta and beta waves observed during REM versus NREM sleep. During sleep in insomnia patients, the relative power of theta and alpha waves was significantly elevated. Increased relative power of alpha waves indicates heightened cortical arousal activity (Jensen and Mazaheri, 2010), while theta waves are associated with cortical information activation and transmission, potentially influencing memory consolidation and cognitive function (Horváth et al., 1976; Sweeney-Reed et al., 2016).

### 4.2 Differences in serological indicators between short-sleep insomnia patients and controls

Sleep is dynamically regulated by multiple neurotransmitters. Serotonergic neurons in the raphe nuclei and orexin cells in the posterior lateral hypothalamus near the fornix participate in sleep–wake state regulation, primarily promoting wakefulness (Monti, 2011). MT, secreted by the pineal gland, promotes sleep. BDNF production is concentrated in regions including the cerebral cortex, hippocampus, and basal ganglia. Its expression correlates closely with slow wave activity (SWA) during NREM sleep and may consolidate deep sleep by regulating synaptic plasticity (ElGrawani et al., 2024).

This study demonstrated significantly elevated serum levels of 5-HT, BDNF, and OXA in the insomnia group. An hypothesis posits that daytime 5-HT release induces the synthesis of hypnagogic factors (Jouvet, 1984). Prolonged sleep deprivation may accumulate sleep homeostasis pressure; studies have indicated that 24-h sleep deprivation significantly elevates serum and brain serotonin levels, suggesting that sleep deprivation in short-sleep insomniacs may underlie elevated 5-HT (Sharma et al., 2023). Furthermore, insomnia patients frequently experience comorbid anxiety, while long-term use of SSRIs, a class of anti-anxiety medications, may also elevate serum 5-HT levels. Insomnia may also influence serum 5-HT concentrations by affecting the pathway that regulates enterochromaffin cell secretion of 5-HT via the neuroendocrine system (Liu et al., 2021). BDNF production exhibits synergistic effects with 5-HT secretion (Martinowich and Lu, 2008). Schmitt *et al.* observed elevated BDNF levels in sleep-deprived subjects (Schmitt et al., 2016). Sleep deprivation, as an acute stressor, activates corticosterone receptors, inducing BDNF elevation—a finding corroborated by Gorgulu et al. (2022). This may be one of the reasons for elevated serum BDNF levels in short-sleep insomnia patients. OXA promotes the transition from sleep to wakefulness and is significantly elevated in the insomnia group. Studies have indicated that short-term sleep deprivation is unrelated to OXA changes (Schmid et al., 2009), with OXA secretion potentially linked to the duration of sleep deprivation, the time of blood collection, and dietary conditions (Liu et al., 2022). Qin et al. (2023) found higher serum OXA levels in female insomnia patients compared with controls, consistent with our findings. MT secretion follows a circadian rhythm, decreasing during daytime hours. This study found no significant difference in serum melatonin levels between the insomnia group and the control group.

### 4.3 Differences in gut microbiota between short-sleep insomnia patients and controls

Short-sleep insomnia entails reduced sleep duration, which activates stress systems and induces chronic oxidative stress (Feige et al., 2023; Vgontzas et al., 2013). Insomnia may also disrupt immune function and neuroendocrine system regulation, further compromising the intestinal barrier and increasing intestinal permeability. Characteristic alterations in the gut microbiota of insomnia patients hold significant diagnostic and therapeutic value (Wang Z. et al., 2022). The gut microbiota influences sleep by modulating the release of neuroactive substances and serum immunomodulatory factors (Roth et al., 2021; Shi et al., 2017). Research has demonstrated that modulating the proportion of dominant microbial populations can effectively improve subjective sleep ratings in insomnia patients (Zhang et al., 2023).

Species diversity analysis revealed no significant difference in *α*-diversity between short-sleep insomnia patients and controls. However, significant differences in *β*-diversity were observed, characterised by reduced intergroup variation in community composition within the insomnia cohort. This indicated lower adaptability to environmental changes among insomnia patients.

Regarding species composition, *Firmicutes_A*, *Bacteroidota*, and *Proteobacteria* constituted the dominant phyla at the phylum level. Short-sleep insomnia is highly correlated with impaired intestinal immune barrier function. *Proteobacteria* harbours numerous pathogenic bacteria, exhibiting significantly elevated relative abundance in the insomnia group. Furthermore, the *F/B* (*Firmicutes*/*Bacteroidota*) ratio decreases in the insomnia group. This reduced *F/B* ratio resembles the gut microbiota structure observed in *NOD2* knockout mice, a pattern associated with increased risk of intestinal inflammation, compromised intestinal barrier function, and heightened intestinal permeability (Sun et al., 2023). At the genus level, *Klebsiella* was an opportunistic pathogen, which contributes most significantly to the compositional differences between the insomnia and control groups. Alterations in gut microbiota abundance can regulate the production of short-chain fatty acids (SCFAs). SCFAs have the effects of protecting intestinal mucosa, promoting gastrointestinal peristalsis and anti-inflammation, which plays a crucial role in the interaction between insomnia and the gut microbiota (Al Nabhani et al., 2016). The abundance of *Firmicutes_A* was lower in the insomnia group than in the control group. *Firmicutes* harbours genes for fermenting dietary fibre, aiding in SCFAs production and interacting with the intestinal mucosa (Baranwal et al., 2023). At the genus level, *Megamonas* and *Roseburia* were significantly elevated in the control group. *Megamonas*, belonging to the *Firmicutes* phylum, produces SCFAs such as acetate and propionate, while *Roseburia* promotes butyrate production. Insomnia can interact with the gut microbiota through the neuroendocrine system. The relative abundances of *Evtepia*, *Anaerostipes*, and *Blautia_A* are elevated in the insomnia group. Within the *Evtepia* taxon, *Evtepiagabavorous* possesses the capacity to consume intestinal GABA. GABA metabolised by gut microbiota can be transmitted to the brain via the vagus nerve, disrupting sleep rhythms (Liang et al., 2022). More often than not, the interaction between insomnia and the gut microbiota is the result of the combined effects of multiple systems. *Anaerostipes* is a butyrate-producing bacterium (Bui et al., 2021; Strandwitz et al., 2019). Research has indicated that insomnia-prone mice exhibit reduced butyrate levels in their intestines. Elevating butyrate concentrations in the gut can promote sleep by modulating orexin neuron activity (Holmberg et al., 2024). A study by Park et al. (2025) also found that elevated abundance of *Anaerostipes* was associated with more severe sleep disturbances and inadequate vitamin C intake. Furthermore, studies have found that the relative abundance of *Anaerostipes* increases following fecal microbiota transplantation therapy in insomnia patients and correlates positively with treatment efficacy (Wong-Chew et al., 2022). Studies have found that *Anaerostipes* improves low-grade intestinal inflammation in patients by promoting butyrate production, which further suggests that the increased abundance of this genus may be related to the self-negative feedback regulation of internal inflammatory level and sleep quality associated with chronic sleep deprivation in patients with short-sleep insomnia (Shao et al., 2023). Research has indicated that the abundance of *Blautia* was significantly increased in patients with sleep disorders, consistent with the findings of the present study (Smith et al., 2019; Zhang et al., 2025), and in another study, *Blautia* was found to be associated with shorter nighttime sleep duration and reduced sleep efficiency (Wang Y. et al., 2022). *Blautia* is a acetate-producing bacterium (Bui et al., 2021; Strandwitz et al., 2019). Acetate produced by gut microbiota in the mouse colon can cross the blood–brain barrier, promoting GABA secretion (Wang et al., 2024). This suggests that the increased relative abundance of *Blautia* in patients with insomnia may be associated with levels of the inhibitory neurotransmitter GABA. Alterations in the gut microbiota associated with insomnia also may be linked to anxiety and depressive emotions comorbid with insomnia. *Dialister* exhibited significantly reduced relative abundance in the insomnia group. It was found that the insomnia patients treated with the oral sedative-hypnotic drug *Ziziphi Spinosae* (ZSSS) showed a significant increase in the abundance of *Dialister*, indicating a correlation between the elevation of *Dialister* and the improvement of sleep quality (Cui et al., 2025), which is consistent with the conclusions drawn from this study. Low *Dialister* levels have been found to associate with accumulated depressive symptoms in insomnia patients (Li et al., 2020).

The ROC curve indicated that *Blautia_A* demonstrated the highest diagnostic accuracy for insomnia at 66.1%, suggesting it may serve as a specific biomarker for short-sleep insomnia.

Associative network analysis revealed that, compared with the control group, the gut microbiota of insomnia patients exhibited significantly looser and more unstable collaborative relationships overall. This unstable state may induce gut barrier disruption and increased intestinal permeability, exacerbating insomnia by influencing the release of sleep-related neurotransmitters via the gut-brain axis. Future research seeking novel therapeutic targets for sleep disorders should focus on microbiota network reconstruction. *Gemmiger_A* played a crucial role in the control group, with its abundance fluctuations potentially influencing the microbial community composition of the controls.

At the functional level, the L-methionine salvage cycle III and S-methyl-5-thio-alpha-D-ribose 1-phosphate degradation pathways represented differentially expressed metabolic pathways between the insomnia and control groups. These pathways were significantly elevated in the control group and are closely associated with species such as *Bacillus*. *Bacillus* is a Gram-positive bacterium capable of producing antibacterial substances, which are beneficial for the development of the intestinal immune system (Frost et al., 2014). The differences in metabolic pathways between the insomnia and control groups may be associated with the inheritance of insomnia-related risk genes, increased susceptibility to insomnia-induced diseases, and adverse outcomes. Upstream and downstream of the L-methionine salvage cycle III, metabolites such as L-glutamine, spermidine and phosphate are generated. In this pathway, the production of *Bacillus* is highly correlated with genes *speE-G275* and *mteK BSU30550*. S-Adenosylmethionine (SAM) is involved in the metabolic pathway of L-methionine salvage cycle III, and SAM provides the methyl required for the conversion of serotonin to MT (Xing and Tu, 2025). It was found that SAM may contribute to the amelioration of advanced sleep phase disorder and its circadian rhythm disorders (Fukumoto et al., 2022), suggesting that this metabolic pathway may regulate sleep by intervening in the methylation cycle. Spermidine is a product of L-methionine salvage cycle III, and Zwighaft et al. (2015) found that a decline in spermidine metabolism was associated with prolonged circadian rhythm time, and that spermine supplementation reversed this type of sleep disorder, suggesting that the decline is associated with sleep disruption. Further research is required to analyse the role of L-methionine salvage cycle III metabolites in hypersomnia-type insomnia and elucidate how gut microbiota influence insomnia.

### 4.4 Correlation between polysomnographic parameters, serological markers, and gut microbiota in insomnia patients

Serum sleep biomarkers exhibited strong correlations with sleep parameters. Partial correlation analysis reveals a significant negative correlation between MT and SL. Oral administration of exogenous MT mimics endogenous MT release, which effectively shortens SL in insomnia patients (Cardinali et al., 2012), which is consistent with the conclusion that there was a significant negative correlation between MT and SL. OXA levels showed a significant negative correlation with SE. Previous studies have indicated that orexin neurons promote wakefulness maintenance by inhibiting sleep-promoting inhibitory neurons, and orexin receptor antagonists effectively enhance SE in insomnia patients (De Luca et al., 2022; Rosenberg et al., 2019). Reduced SE in insomnia patients may represent a self-regulatory mechanism involving suppression of orexin neuron activity and decreased OXA levels. Research has indicated that reduced SL is dose-dependent with daridorexant (a dual orexin receptor antagonist) (Dauvilliers et al., 2020), which is consistent with the conclusion that OXA levels were positively correlated with SL. OXA levels also showed a significant positive correlation with the relative power delta waves during REM sleep, suggesting elevated OXA may reflect insomniacs’ heightened need for deep cerebral rest.

Spearman’s partial correlation analysis of insomnia patients with established microbiota and serum samples revealed a significant negative correlation between OXA and *Anaerostipes*. *Anaerostipes* is a butyrate-producing bacterium. Research has demonstrated that increasing butyrate levels in the mouse gut modulates orexin neuron activity to promote sleep, consistent with the findings of this study (Wang et al., 2024). Previously mentioned increase in *Anaerostipes* may be associated with activation of negative feedback mechanism related to long-term sleep deprivation, the premise of which may lie in the fact that dysbiosis or osmotic up-regulation of inflammatory factors such as lipopolysaccharide in the gut leads to the induction of the production of ceramides, which can directly inhibit OXA (Anderson et al., 2019). Additionally, the release of OXA is accompanied by activation of the sympathetic nervous system, and this activation is one of the triggers for increased inflammation in the intestinal microbiota, and increased inflammation in the intestinal tract may also be associated with the reverse increase in *Anaerostipes*, the beneficial gut bacteria associated with SCFAs (Anderson et al., 2019).

Spearman’s partial correlation analysis in insomnia patients with complete microbiota and polysomnographic data revealed a positive correlation between *Anaerostipes* and the relative power of theta and beta waves during NREM sleep (*p* — 0.01). This suggests that increased *Anaerostipes* abundance in insomnia patients may indicate excessive cortical arousal, indirectly reflecting a lowered sleep threshold during NREM sleep. *Dialister* exhibited a significant negative correlation with N3 sleep and its proportion (*p*—0.01). Its reduced relative abundance in the insomnia group, coupled with this negative correlation, may indicate impaired deep sleep consolidation. RDA simultaneously considers the combined effects of multiple environmental factors on target variables. Results indicated that N3 sleep and SL significantly influence the distribution of gut microbiota markers in both the insomnia and control groups.

## 5 Limitations of the study

This study also has certain limitations. Firstly, serum markers are easily affected by factors such as blood collection time and dietary habits, and serum neurotransmitter levels cannot fully reflect central nervous system activity. However, central serum is difficult to collect, so future studies may integrate cerebrospinal fluid testing or functional imaging for deeper investigation, and further validation using animal models is necessary. Secondly, while strict exclusion criteria for digestive system disorders were applied during sample collection to mitigate dietary and gastrointestinal influences on gut microbiota, potential confounding effects cannot be entirely ruled out. Dietary habits have a significant impact on the composition of the gut microbiota. Although this study confirmed no significant difference in BMI between the insomnia group and the control group (*p* > 0.05), this cannot fully account for the effects of dietary habits. Regarding medication use among participants, this study excluded those with major chronic conditions such as diabetes, coronary heart disease, and hyperthyroidism. However, it cannot completely rule out the potential impact of participants concealing their history of chronic disease medication use. As this study is a real-world study, it was impossible to completely prevent insomnia patients from self-administering insomnia medications beforehand, and the correlation analysis in this study has controlled for medication-related bias. Insomnia patients were prohibited from taking oral insomnia medications prior to monitoring, which may have caused withdrawal reactions. Other lifestyle factors that may affect the gut microbiota, such as exercise habits, were not strictly controlled and also constitute limitations of the study. Future research should develop corresponding animal models to validate clinical findings. Furthermore, regarding the identification of gut microbiota biomarkers, future work should establish the modulatory effects of altering gut microbiota composition on short-sleep insomnia through foundational experiments. The underlying mechanisms of gut microbiota in insomnia should then be further validated via interventional studies such as microbiota transplantation. Due to the small sample size, the stability and generalizability of the results from RDA and correlation analyses are somewhat limited; therefore, this study is primarily intended for exploratory analysis. In the future, it will be necessary to expand the sample size and validate the results using further predictive modelling methods to enhance the robustness of the conclusions. Additionally, the complex influencing factors of gut microbiota and the incomplete inclusion of confounding variables may affect the stability of the results, necessitating future validation through animal experiments.

## 6 Conclusion and outlook

This study represents a real-world investigation of sleep-related biomarkers in insomnia patients, revealing significant differences in polysomnographic parameters, serum biomarkers, and gut microbiota between short-sleep insomnia and healthy controls. Correlations exist between gut microbiota and sleep parameters and serum factors, suggesting targeted gut microbiota modulation may improve insomnia. However, future large-scale studies are required to elucidate and validate the specific mechanisms underlying gut microbiota-insomnia interactions.

In terms of potential clinical future applications, SCFAs may influence sleep by stimulating gut hormone release, modulating neuronal pathways via bile acid binding, and inducing tryptophan hydroxylase1 (Tph1) expression (Dicks, 2022; Yang X. et al., 2023). Future studies should explore how insomnia-associated microbiota regulates the association between sleep and gut microbiota by affecting the abundance of SCFAs. Correlation analyses between neurotransmitters and gut microbiota suggest that it is promising to regulate gut microbiota to modulate the release of sleep-related neurotransmitters in insomnia patients, so as to improve the sleep quality. In addition, this study innovatively correlates polysomnographic parameters with gut microbiota, providing a novel insight for further intervening gut microbiota via FMT or pharmaceuticals to reduce the number of spontaneous awakenings and prolong total sleep time in insomnia patients. Previous studies on insomnia improvement mostly focused on the improvement of subjective sleep parameters, while this study indicates that sleep improvement can be observed and even predicted by monitoring polysomnographic parameters, electroencephalogram frequencies, the release of sleep-related neurotransmitters, and even changes in differential strains.

## Data Availability

The data presented in this study can be found in the NCBI Sequence Read Archive (SRA), under accession PRJNA1506090 (https://www.ncbi.nlm.nih.gov/sra/PRJNA1506090).

## Footnotes

1
https://cloud.oebiotech.cn/spa#/home
